# Cerebrospinal fluid profile in coronavirus disease 2019, a prospective case series study

**DOI:** 10.7150/ijms.111747

**Published:** 2025-09-08

**Authors:** Huiying Lin, Xiaoli Zhang, Hangfeng Li, Shuangfang Fang, Huapin Huang, Nan Liu, Houwei Du

**Affiliations:** 1 Department of Neurology, Fujian Medical University Union Hospital, Fuzhou, China.; 2 Clinical Research Center for Precision Diagnosis and Treatment of Neurological Diseases of Fujian Province, Fuzhou, China.; 3 Department of Laboratory Medicine, Fujian Medical University Union Hospital, Fuzhou, China.; 4 Department of Neurology, Longyan First Hospital, Longyan, China.; 5 Department of Rehabilitation Medicine, Fujian Medical University Union Hospital, Fuzhou, China.; 6 Institute of Clinical Neurology, Fujian Medical University, China.

**Keywords:** coronavirus disease 2019, severe acute respiratory syndrome coronavirus type 2, nervous system disease, cerebrospinal fluid, neurological involvement

## Abstract

**Background:** Cerebrospinal fluid (CSF) analysis in patients with Coronavirus disease 2019 (COVID-19) and co-existing acute neurological involvement remains poorly understood.

**Objective:** To investigate the CSF profile in patients with COVID-19 and co-existing acute neurological involvement.

**Methods:** This prospective case series study included patients with confirmed severe acute respiratory syndrome coronavirus type 2 (SARS-CoV-2) infection and co-existing acute neurological involvement who underwent lumbar puncture in two teaching hospitals between November 2022 and April 2023. Demographics, clinical characteristics, and CSF profile, including leukocyte count, total protein, and glucose levels, oligoclonal band (OCB) patterns, blood-CSF barrier function, SARS-CoV-2 mRNA, and SARS-CoV-2 antibodies were described.

**Results:** A total of 26 participants were analyzed. The median age was 51 (interquartile range [IQR] 39-76) years, and 18 (69.2%) were male. The median open CSF pressure was 140mm (IQR 110-183) water column, and the median CSF total protein was slightly elevated (485 [IQR 350-611] mg/L). The most frequent pathological finding was elevated CSF total protein (12 [46.2%] samples) and blood-CSF barrier dysfunction (12 [46.2%] samples). SARS-CoV-2 was undetectable in all CSF samples using the reverse-transcriptase-polymerase-chain-reaction detection. SARS-CoV-2-IgG-antibody was positive in five CSF samples, while SARS-Cov-2 IgM antibodies were not detected in all participants.

**Conclusions:** This study showed that some patients with COVID-19 and co-existing acute neurological involvement presented non-specific inflammatory CSF abnormalities despite no SARS-CoV-2 being detected in the CSF. Our findings suggest that neurological injury is related to disordered immune responses associated with systemic inflammation rather than direct virus invasion.

## Introduction

The global outbreak of the coronavirus severe acute respiratory syndrome coronavirus 2 (SARS-CoV-2) has killed millions of people worldwide 1,2. There is accumulating evidence of central neurological symptoms and conditions seen in the context of coronavirus disease 2019 (COVID-19), including headache, encephalopathy, seizures, stroke, Guillain-Barré syndrome, and acute disseminated encephalomyelitis 3-6. Cytokine storm or neurological verification in cerebrospinal fluid have been reported, but it is unknown whether it is caused by direct viral infection or immune response 6.

The COVID-19 pandemic highlighted the existence of neurological symptoms associated with SARS-CoV-2 infection and raised the question of the neuropathogenicity of coronaviruses 7. Few case reports have identified SARS-CoV-2 in the CSF by quantitative reverse transcriptase polymerase chain reaction (qRT-PCR) or sequencing techniques 8. However, a comprehensive study of 150 lumbar punctures in 127 patients with COVID-19-associated neurological disease showed that although inflammatory changes were common, direct detection of SARS-CoV-2 in CSF was rare 5. CSF biomarkers can characterize the central nervous syndrome response to infection by directly detecting invading pathogens and host inflammatory responses 9. To date, comprehensive data on the cerebrospinal fluid (CSF) profile in patients with COVID-19 and co-existing acute neurological involvement remains not well understood. In this prospective observational study, we aimed to describe the CSF profile in patients with acute neurological diseases associated with COVID-19.

## Methods

### Participants

This prospective case series study included consecutive adult patients (i.e., 18 years or older) with confirmed SARS-CoV-2 infection who underwent lumbar puncture for CSF testing due to co-existing acute neurological involvement in two teaching hospitals during the Omicron epidemic in mainland China, between November 2022 and April 2023. Patients with pre-existing inflammatory or cerebrovascular conditions were excluded since these conditions may cause CSF changes and are considered less likely related to COVID-19. Confirmed SARS-CoV-2 infection was defined by the detection of SARS-CoV-2 mRNA in nasopharyngeal swabs using qRT-PCR detection, with a cycle threshold value of 37 or lower 10.

### Data collection

Demographics, clinical characteristics, indications for lumber puncture, and CSF parameters were collected through the electronic medical database.

### CSF assessment

CSF samples were immediately processed to determine cell counts, total protein, and glucose levels. SARS-CoV-2 mRNA in CSF samples was detected using the RT-qPCR kit (Zhengzhou Autobio, China). Detection of oligoclonal bands (OCB) in the CSF was performed by isoelectric focusing on comparing paired CSF and serum samples. Oligoclonal IgG bands were assessed by isoelectric focusing and evaluated according to an international consensus. Immunoglobulins and albumin were measured immunonephelometrically 11. Oligoclonal bands (OCB) patterns were classified as type 1, OCB detected in neither CSF nor serum; type 2, oligoclonal IgG bands detected in only CSF; type 3, OCB in CSF and serum with additional bands in CSF; type4, identical OCB in CSF and serum; and type 5, monoclonal bands in CSF and serum. Types 2 and 3 indicate intrathecal IgG synthesis, type 4 indicates a systemic, ongoing inflammatory process, and type 5 indicates systemic paraproteinemia 3.

### Evaluation of blood-CSF barrier function

Blood-CSF barrier function was assessed using the CSF/serum albumin quotient: QAlb = AlbCSF [mg/L]/Albserum [g/L]. As the upper reference limit of QAlb is age-dependent, Qlim (Alb) was calculated as (4+[a/15])×10^-3^, with a representing the patient's age. Dysfunction of the blood-CSF barrier was defined as QAlb > Qlim (Alb) 12.

### Statistical analyses

Continuous variables were summarized as mean with standard variations if normally distributed or median with interquartile range (IQR) if not normally distributed. Categorical variables were expressed as frequencies with percentages. Spearman's rho test was used to assess correlations. *P* values < 0.05 were considered statistically significant. All analyses were conducted using SPSS Statistics Version 25.0 (IBM, Chicago, IL, USA).

## Results

We included 26 consecutive patients with confirmed SARS-CoV-2 infection who received lumbar puncture due to co-existing acute neurological involvement in the final analysis. The median age was 51 (IQR 39-76), and 18 (69.2%) were male. **Table 1** summarizes the demographics and clinical characteristics. The most commonly reported neurological symptoms were limb weakness or numbness (12 cases [46.2%]), followed by dizziness (10 cases [38.5%]), headache (8 cases [30.8%]), and impaired consciousness (8 cases [30.8%]). The clinical diagnosis of neurological involvement included Herpes Simplex encephalitis (2 cases [7.7%]), Streptococcus pneumoniae encephalitis (1 case [3.8%]), encephalitis with unknown etiology (4 cases [15.4%]), acute myelitis (2 cases [7.7%]), and acute cerebellitis (2 cases [7.7%]), cranial neuritis (2 cases [7.7%]), stroke (7 cases, [26.9%]), Guillain-Barre syndrome (3 cases [11.5%]), demyelinating encephalopathy (1 case [3.8%]), epilepsy with unknown etiology (1 case [3.8%]), extrapyramidal disorder with unknown etiology (1 case [3.8%]).

**Table 2** lists the significant findings of the CSF profile. The median open CSF pressure was 140mm [IQR 110-183] water column, the median leucocyte count was 2×10^6^/L, and the median CSF total protein level was 485 mg/L (IQR 350-611). Six (23.1%) cases had elevated open CSF pressure (ranging from 195 to 350 mmH_2_O), 6 (23.1%) had elevated leucocyte counts (ranging from 19 to 225×10^6^/L), and 12 (46.2%) of our participants had increased CSF total protein (ranging from 491 to 803 mg/L).

**Figure 1:**
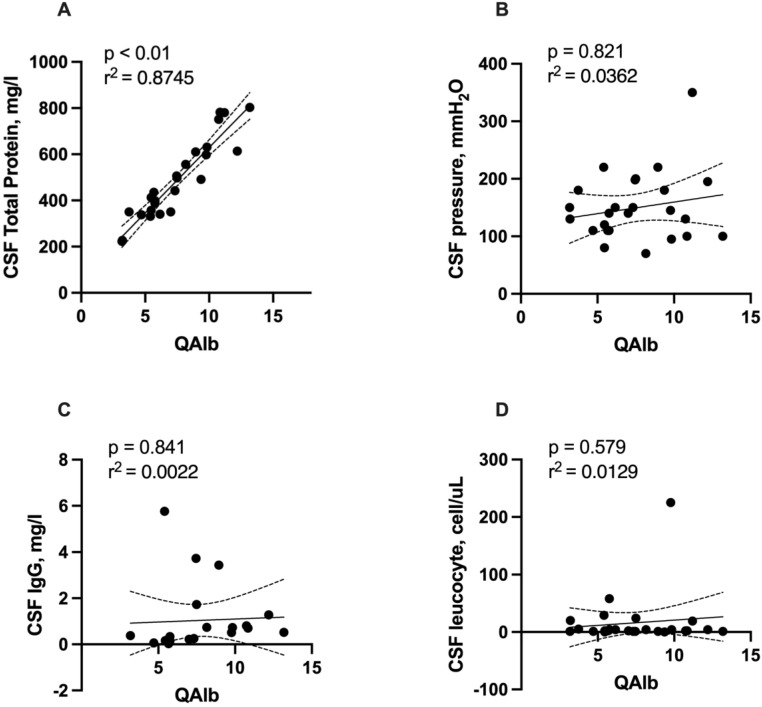
** Correlation of QAlb with total protein concentration, opening CSF pressure. CSF IgG, and leucocyte counts.** Abbreviations: CSF = cerebrospinal fluid; QAlb = AlbCSF[mg/L]/Albserum[g/L]

**Figure 2:**
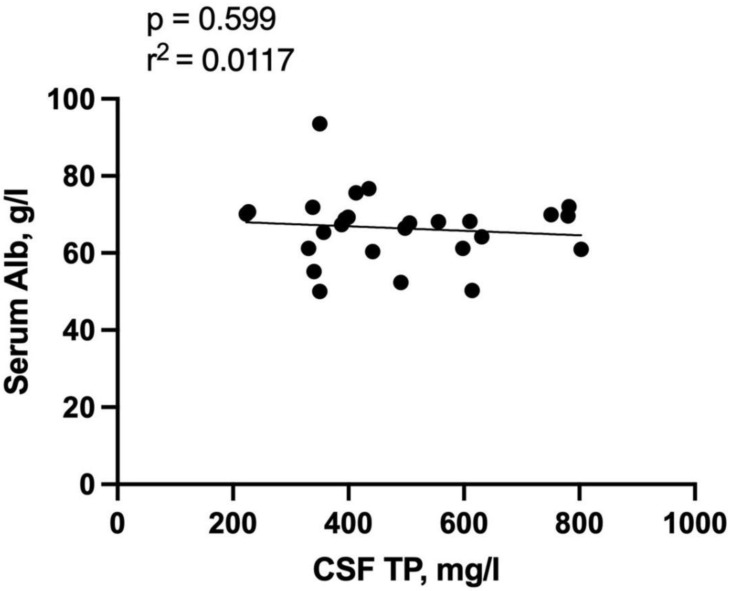
** Correlation of Serum Albumin with CSF total protein concentration.** Abbreviations: CSF = cerebrospinal fluid; TP = total protein.

Elevation of QAlb, a sign of disruption of the blood-CSF barrier, was found in 12 (46.2%) of patients. Patients with blood-CSF barrier dysfunction had a median QAlb of 7.45 (IQR 5.52-9.67; range: 3.19-13.19). Total protein concentration was significantly correlated with QAlb (r = 0.926, r^2^ = 0.8745, p < 0.001, **Figure 1A**). No significant correlation was detected of QAlb with opening CSF pressure, CSF IgG, and leucocyte counts. (**Figure 1B, 1C, 1D**). We detected no significant association between CSF albumin concentration and serum albumin levels using Spearman's rho test (r=0.0117, p = 0.599, **Figure 2**).

SARS-Cov-2 IgM antibody was undetectable in all CSF samples, while SARS-CoV-2 IgG antibodies were positive in 5 (19.2%) patients (Cases No. 5, 13, 16, 17, 20). Two of these five patients with positive SARS-CoV-2 IgG antibody presented blood-CSF barrier dysfunction.

**Table 3** shows the percentage of abnormal CSF parameters in different central nervous system disorders. Patients were categorized into four groups of clinical syndromes to overcome the small number of cases: (1) inflammatory neurological diseases, which included encephalitis, myelitis, cerebellitis, and cranial neuritis; (2) stroke; (3) Guillain-Barré Syndrome; and (4) other diseases including extrapyramidal disorder, demyelinating encephalopathy and epilepsy. Among 13 patients with inflammatory neurological disease, 5 (38.5%) presented with increased CSF leucocyte counts, 6 (46.2%) had elevated CSF total protein concentrations and blood-CSF barrier dysfunction, and 4 (30.8%) patients presented elevated CSF pressure. In seven patients with stroke, 3 (42.9%) indicated elevated CSF total protein levels, 2 (28.6%) had increased CSF pressure and blood-CSF barrier dysfunction, and 1 (14.3%) showed elevated CSF leukocyte counts. Among the three patients with Guillain-Barré Syndrome, all exhibited elevated CSF total protein levels and blood-CSF barrier dysfunction. Among three cases with other syndromes, only one (33.3%) exhibited increased CSF total protein concentrations and blood-CSF barrier dysfunction **(Table 3)**.

Data regarding the OCB patterns in the CSF and serum samples were available in 17 out of the 26 participants. Of these 17 CSF samples, only two (11.8%) were classified as type 2, indicating intrathecal synthesis (**Table 4**). Among these two patients who showed intrathecal synthesis, only one was detected positive for SARS-CoV-2 IgG.

**Table 5** lists the peripheral blood profiles of our patients. Serum IgG antibodies against SARS-CoV-2 were detected in 23 out of the 26 participants, and co-existent SARS-CoV-2 IgM were detected in seven participants. Among these 23 patients with serum SARS-CoV-2 IgG, the median albumin level was 68.0 [IQR 61.2-70.1] g/L, the median leucocyte count was 7.3×10^6^/L, and the median glucose level was 5.2 [IQR 4.5-6.0] mmol/L. A total of 21 (91.3%) patients had elevated albumin (ranging from 55.2 to 93.6 g/L), seven (30.4%) had elevated leucocyte counts (ranging from 10.1 to 14.6×10^9^/L), and six (26.1%) patients had increased glucose (ranging from 6.6 to 9.8 mmol/L).

SARS-CoV-2 IgG in the CSF was detected in 23 (88.5%) patients, including 15 patients with SARS-CoV-2 infection only and 8 patients with other viral co-infections. As shown in Table 6, patients with SARS-CoV-2 infection only had similar proportion of elevated CSF pressure (5 [23.3%] vs. 2 [25.0%], p = 0.679), elevated total protein level (7 [46.7%] vs.4 [50.0%], p = 0.879) and blood-brain barrier disruption (8 [53.3%] vs. 4 [50.0%], p = 0.879) compared to those with co-infections. Elevated leukocyte count (2 [13.3%] vs. 3 [37.5%], p = 0.181) and IgG detection (3 [20.0%] vs. 1 [12.5%], p = 0.651) were more frequently seen in patients with SARS-CoV-2 infection only compared to those with other infections, but the differences did not reach statistical significance.

## Discussion

The present study yielded several important findings. First, some COVID-19 patients with co-existing neurological disorders may present abnormal CSF profiles despite SARS-CoV-2 mRNA were not detected in the CSF. The most frequent pathological finding was elevated CSF total protein and blood-CSF barrier dysfunction. Moreover, some of the patients tested positive for SARS-CoV-2 IgG antibodies in their CSF, while none tested positive for SARS-CoV-2 IgM antibody. Our findings suggest that CSF analysis is useful in the clinical evaluation of these patients and may contribute to a better understanding of the neurological involvement associated with COVID-19.

In agreement with our findings, another small cohort showed that although SARS-CoV-2 was not detected in CSF samples by qRT-PCR, many patients presented abnormal CSF profiles 8. Our findings that six (23.1%) cases had elevated open CSF pressure were supported by several previous case series studies that showed open CSF pressure ranged between normal and slightly elevated values in most cases 13. Moreover, a retrospective study showed that one-third of patients had high open CSF pressure, although this characteristic was not associated with a specific neurological picture 1. Our data showed that some COVID-19 patients with co-existing neurological disorders presented pleocytosis. In line with our findings, pooled data showed that among 409 patients with central nervous system disorders who had available data of CSF white blood cell count, 29 (7%) had a CSF leukocyte count of 21-100 cells/μL, and 8 (2%) had a CSF leukocyte count of 100 cells/μL or higher 14. Our data that nearly half of our participants had elevated CSF total protein concentration (>450 mg/L) was supported by pooled observational data showing that among 397 patients who had available data on CSF total protein, 160 (40%) of whom had increased CSF total protein levels 11. The elevation of CSF total protein levels in our patients can be partly explained by the blood-CSF barrier dysfunction since we detected a significant correlation between CSF total protein concentration with QAlb.

Mechanisms implicated in the above-mentioned abnormal CSF findings remain uncertain but might include neuronal injury associated with direct virus infection, a para- or post-infectious inflammatory disease, or a secondary process due to severe effects of a systemic disorder 15,16. Our study showed that SARS-Cov-2 mRNA was undetectable using the qRT-PCR technology in all CSF samples. Similar to our findings, data from Sweden showed undetectable SARS-CoV-2 mRNA in all 31 CSF samples 17. Moreover, a case series study showed that patients with COVID-19 and neurological disorders had undetectable or extremely low levels of SARS-CoV-2 mRNA in the CSF 1. A prospective observational study of pregnancy women indicated no evidence of SARS-CoV-2 in the CSF of COVID-19 patients with early neurological symptoms.

Therefore, the neurological symptoms may not be caused by the direct damage of the virus to the central nervous system 18. The findings of the above-mentioned studies may suggest a low likelihood of direct viral involvement in neurological symptoms. Of note, the methodology used for SARS-CoV-2 detection in the CSF may not have been sensitive enough to get low viral loads, leading to potential underestimation of viral involvement. Given that COVID-19 patients with neurologic involvement may present a wide range of clinical manifestations, the neuroinvasion potential of SARS-CoV-2, neuroinflammation as a possible driver, and the connection with blood-brain barrier disruption regardless of intrathecal inflammation should be more investigated in future studies 5,19.

Our data showed that the SARS-CoV-2 IgM antibody was undetectable in all CSF samples, while the SARS-CoV-2 IgG antibody was detected in 5 (19.2%) patients. Two of these five patients with SARS-CoV-2 IgG antibody presented blood-CSF barrier dysfunction. The findings aforementioned may prevent direct conclusions over the active destruction of SARS-CoV-2 infection in the CSF. The detectable SARS-CoV-2 IgG antibodies in some cases may be associated with previous virus exposure or vaccination 20. Moreover, high levels of circulating inflammatory cytokines after SARS-CoV-2 infection may possibly disrupt the blood-CSF barrier allowing for antibodies and other inflammatory mediators to enter the brain parenchyma 21. Thus, SARS-CoV-2 antibodies in the CSF of COVID-19 patients possibly reflect blood-CSF barrier disruption associated with systemic inflammation. Although we did not have data available to assess antibody-index, a previous study with antibody-index calculations did not support a direct SARS-CoV-2 brain infection 22. Whether the SARS-CoV-2 antibodies detection may have diagnostic and prognostic implications needs to be investigated in future studies.

Our data showed that two patients (11.8%) had the type 2 OCB pattern consistent with a possible intrathecal IgG synthesis. Similar to our findings, data from Germany showed that 5/83 (6%) patients with post-COVID-19 syndrome presented CSF-restricted OCB (pattern 2 in 4/83 [5%], pattern 3 in 1/83 [1%]) 23. Moreover, a case series study showed that CSF-restricted OCB was detected in 11.1% (5/45) of patients with neuroimmune complications of COVID-19 24. Further studies are required to validate these findings, contributing to better elucidating the pathophysiological mechanisms.

Comparative analysis of CSF samples from patients with COVID-19 infection only and those with co-existing other infections showed no statistically significant differences in CSF profiles (e.g., leukocyte count, total protein, and open CSF pressures). This finding suggests that the neurological changes observed in the CSF may not be solely attributed to COVID-19 and could potentially be influenced by other underlying infections. This issue needs to be investigated in future studies.

Our finding should be interpreted with caution due to its small size, which may not enough represent the broader population of patients with COVID-19 and neurological complications. Although comparing CSF characteristics of general COVID-19 infected patients to neurological involvement COVID-19 patients may better demonstrate our conclusions, it is not feasible to collect CSF of general COVID-19 patients without neurological involvement in clinical practice. Moreover, comparing the peripheral blood characteristics of COVID-19 individuals with and without neurological involvement may better observe the changes caused by neurological injury or a possible connection with the blood-brain barrier. However, only including COVID-19 patients with neurological involvement in this study does not allow us to make these comparisons, which needs to be investigated in future studies. Notably, the timing of lumbar puncture and the time of COVID-19 infection were uneven among our patients, which may affect our findings. *‌*Beyond routine hematological parameters, these comparisons could include biomarkers associated with inflammatory responses and blood-brain barrier function, such as cytokines, chemokines, and blood-brain barrier-specific proteins. Appropriate statistical methods will be employed to identify differentially expressed markers in patients with neurological involvement and to analyze the relationship between these alterations and neural injury or blood-brain barrier dysfunction. This approach aims to further elucidate the patho-physiological mechanisms underlying neurological involvement in COVID-19 patients. Moreover, some CSF parameters, such as concentration of total Tau, neurofilament light chain proteins 25, and inflammatory index, including CD4/CD8, cytokines 26, the cellularity of B cells, T-subsets of lymphocytes, the activation of lymphocytes, and other cells (e.q.,neutrophils) were not routinely measured in our patients due to Medicare policy. A multicenter study of 150 lumbar punctures in 127 COVID-19 patients with neurological symptoms showed that cytokine levels were frequently elevated in the CSF (often associated with blood-brain barrier dysfunction), partly remaining positive at high levels for weeks to months 5. Also, we only analyzed Chinese participants; whether our findings may be generalizable to other populations needs to be validated in future studies. Lastly, the lack of longitudinal data does not allow us to determine these CSF changes over time.

## Conclusion

This study showed that some patients with COVID-19 and co-existing neurological involvement may present non-specific inflammatory CSF abnormalities despite no SARS-CoV-2 being detected in the CSF. Our findings suggest that neurological injury is related to misdirected immune responses associated with systemic inflammation rather than direct virus invasion.

## Figures and Tables

**Table 1:** Demographics and clinical characteristics of 26 participants.

Cases	Sex	Age	COVID-19 vaccine doses	Day from COVID-19 to lumber puncture	Hyper-tension	Diabetes	Cardio-cerebrova-scular disease	Chronic kidney disease	Limb numbness	Dizziness	Headache	Impaired consciousness	Clinical diagnosis
1	Male	51	3	8	No	No	No	No	Yes	No	No	No	Guillain-Barre syndrome
2	Male	24	3	18	No	No	No	No	Yes	Yes	No	No	Acute myelitis
3	Male	35	3	34	No	No	No	No	Yes	No	No	No	Cerebral infarction
4	Male	76	2	24	Yes	No	No	No	No	Yes	Yes	No	Encephalitis with unknown etiology
5	Male	85	1	28	No	No	No	No	Yes	No	No	No	Cerebral infarction
6	Female	52	2	43	No	No	No	No	Yes	No	No	No	Acute myelitis
7	Male	75	3	32	No	No	No	No	Yes	Yes	No	No	Guillain-Barre syndrome
8	Male	80	2	7	No	No	No	No	No	No	No	No	Extrapyramidal disorder
9	Male	64	3	11	No	No	No	No	No	No	No	No	Acute cerebellitis
10	Male	16	3	24	No	No	No	No	No	Yes	Yes	Yes	HSV-7 virus encephalitis
11	Female	60	2	23	No	No	No	No	No	No	Yes	No	Cranial neuritis
12	Male	40	3	37	No	No	No	No	No	No	Yes	Yes	Encephalitis with unknown etiology
13	Male	51	2	10	No	No	No	No	No	Yes	Yes	No	Streptococcus pneumoniae encelphaltis
14	Male	51	3	13	Yes	No	No	No	No	No	No	No	Brainstem infarction
15	Female	79	3	88	Yes	Yes	Yes	Yes	Yes	Yes	Yes	Yes	Cerebral infarction
16	Male	76	1	97	Yes	No	No	No	No	Yes	Yes	No	Acute cerebellitis
17	Male	47	2	NA	Yes	No	No	No	No	No	No	No	Cranial neuritis
18	Female	19	3	108	No	No	No	No	No	Yes	Yes	Yes	Encephalitis with unknown etiology
19	Female	56	3	NA	Yes	Yes	No	No	Yes	No	No	No	Cerebral infarction
20	Female	83	2	3	Yes	No	No	No	Yes	Yes	No	No	Cerebral infarction
21	Female	45	3	6	Yes	No	No	No	No	Yes	No	No	Demyelinating encephalopathy
22	Male	71	3	9	No	No	No	No	No	No	No	Yes	Epilepsy
23	Female	78	3	6	Yes	No	Yes	No	Yes	No	No	Yes	Intracranial venous sinus thrombosis
24	Male	45	3	26	Yes	No	No	No	No	No	No	Yes	Encephalitis with unknown etiology
25	Male	35	3	3	No	No	No	No	Yes	No	No	Yes	HSV-2 virus encephalitis
26	Male	34	3	1	No	No	No	No	Yes	No	No	No	Guillain-Barre syndrome

Abbreviations: COVID-19 = Coronavirus disease 2019; HSV = Herpes Simplex

**Table 2:** CSF profile of included participants.

Cases	CSF pressure (mmHg)	Leukocyte count (cells/μL)	Total protein (mg/L)	Glucose (mmol/L)	IgG (mg/L)	QAlb	Qlim	Blood-CSF barrier dysfunction	SARS-CoV-2 mRNA	SARS-CoV-2 IgG (S/C.O)	SARS-CoV-2 IgG	SARS-CoV-2 IgM	Combined with other virus infections
1	100	1	803	3.95	0.52	13.18	7.4	yes	0	0.52	negative	negative	No
2	150	1	223	4.13	0.38	3.18	5.6	no	0	0.38	negative	negative	No
3	110	4	388	3.33	0.15	5.76	6.3	no	0	0.15	negative	negative	No
4	145	225	598	2.86	0.51	9.77	9.1	yes	0	0.51	negative	negative	Yes
5	195	4	614	3.44	1.28	12.20	9.7	yes	0	1.28	positive	negative	No
6	140	58	399	4.26	0.34	5.76	7.5	no	0	0.34	negative	negative	No
7	95	4	631	4.00	0.73	9.83	9	yes	0	0.73	negative	negative	No
8	110	3	394	3.77	0.08	5.73	9.3	no	0	0.08	negative	negative	No
9	80	0	413	3.62	0.16	5.46	8.3	no	0	0.16	negative	negative	Yes
10	70	4	556	3.35	0.74	8.16	5.1	yes	0	0.74	negative	negative	Yes
11	100	2	782	3.87	0.7	10.85	8	yes	0	0.70	negative	negative	No
12	110	1	338	3.62	0.06	4.70	6.7	no	0	0.06	negative	negative	Yes
13	220	29	331	2.70	5.76	5.41	7.4	no	0	5.76	positive	negative	No
14	130	2	751	2.70	0.8	10.74	7.4	yes	0	0.80	negative	negative	No
15	150	1	442	5.56	0.25	7.32	9.3	no	0	0.25	negative	negative	No
16	198	1	506	3.30	3.73	7.46	9.1	no	0	3.73	positive	negative	Yes
17	220	1	610	3.44	3.44	8.94	7.1	yes	0	3.44	positive	negative	No
18	110	2	435	3.57	0.03	5.67	5.3	yes	0	0.03	negative	negative	Yes
19	140	2	350	4.10	0.22	7.00	7.7	no	0	0.22	negative	negative	No
20	200	24	498	4.47	1.73	7.49	9.5	no	0	1.73	positive	negative	No
21	120	2	357	3.13	0.16	5.46	7	no	0	0.16	negative	negative	No
22	180	0	491	NA	NA	9.37	8.7	yes	0	0.11	negative	negative	No
23	150	4	340	NA	NA	6.16	9.2	no	0	1.56	negative	negative	No
24	130	20	227	NA	NA	3.21	7	no	0	2.01	negative	negative	Yes
25	350	19	780	NA	NA	11.20	6.3	yes	0	0.22	negative	negative	Yes
26	180	5	350	NA	NA	3.74	6.3	yes	0	0.31	negative	negative	No

Abbreviations: CSF = cerebrospinal fluid; QAlb = AlbCSF[mg/L]/Albserum[g/L]; Qlim = (4+[age/15])×10^-3^; SARS-CoV-2 = severe acute respiratory syndrome coronavirus type 2

**Table 3:** Abnormal CSF parameters in different neurological syndromes.

Clinical syndrome	Open CSF pressure >180mmH_2_O	Leucocyte > 5cells/μL	Total protein > 450 mg/L	Abnormal glucose^c^ (mmol/L)	blood-CSF barrier dysfunction	Positive SARS-CoV-2 IgG	Positive SARS-CoV-2 IgM
Inflammatory syndrome^a^ (n = 13)	4 (30.8%)	5 (38.5%)	6 (46.2%)	0	6 (46.2%)	3 (23.1%)	0
Stroke (n = 7)	2 (28.6%)	1 (14.3%)	3 (42.9%)	1 (14.3%)	2 (28.6%)	2 (28.6%)	0
GBS (n = 3)	0	1 (33.3%)	3 (100%)	0	3 (100%)	0	0
Other diseases^b^ (n = 3)	0	0	1 (33.3%)	0	1 (33.3%)	0	0

^a^Inflammatory neurological diseases included encephalitis (n = 7), myelitis (n = 2), cerebellitis (n = 2) and cranial cranial neuritis (n = 2) ^b^Other diseases included extrapyramidal diseases (n = 1), demyelinating encephalopathy (n=1) and epilepsy (n=1) ^c^ Abnormal glucose refers to > 4.5 or < 3.6mmol/L ^d^ Blood-CSF barrier dysfunction was defined as QAlb > Qlim (Alb) Abbreviations: CSF = cerebrospinal fluid; GBS = Guillain-Barré Syndrome; SARS-CoV-2 = severe acute respiratory syndrome coronavirus type 2; QAlb = AlbCSF[mg/L]/Albserum[g/L]; Qlim = (4+[age/15])×10^-3^

**Table 4:** Oligoclonal bands in serum and CSF.

Cases	Oligoclonal bands in serum	Oligoclonal bands in CSF	Oligoclonal patterns	SARS-CoV-2 IgG
1	0	0	Type 1	negative
2	0	0	Type 1	negative
3	/^*^	/	/	negative
4	0	0	Type 1	negative
5	0	1	Type 2	positive
6	/	/	/	negative
7	0	0	Type 1	negative
8	0	1	Type 2	negative
9	0	0	Type 1	negative
10	0	0	Type 1	negative
11	0	0	Type 1	negative
12	0	0	Type 1	negative
13	0	0	Type 1	positive
14	0	0	Type 1	negative
15	0	0	Type 1	negative
16	/	/	/	positive
17	/	/	/	positive
18	0	0	Type 1	negative
19	0	0	Type 1	negative
20	1	1	Type 4	positive
21	0	0	Type 1	negative
22	/	/	/	negative
23	/	/	/	negative
24	/	/	/	negative
25	/	/	/	negative
26	/	/	/	negative

^*^ / = data unavailable Abbreviations: CSF = cerebrospinal fluid; SARS-CoV-2 = severe acute respiratory syndrome coronavirus type 2

**Table 5:** Peripheral blood profile of included participants.

Cases	Leukocyte count (cells/µL)	Hemoglobin (g/L)	Platelet count (cells/µL)	Fibrinogen (g/L)	Glucose (mmol/L)	Albumin (g/L)	SARS-CoV-2 IgG	SARS-CoV-2 IgM
1	5.18	141	238	2.75	4.48	60.93	positive	positive
2	6.21	144	300	3.01	3.34	70.13	positive	negative
3	7.02	140	325	2.41	4.4	67.36	positive	negative
4	5.93	136	226	/	5.74	61.21	positive	negative
5	11.22	115	198	3.83	5.02	50.33	positive	positive
6	8.23	115	292	3.98	6.64	69.27	positive	negative
7	8.8	136	224	2.71	4.73	64.19	positive	negative
8	6.06	141	366	4.65	5.42	68.76	positive	positive
9	5.71	138	181	2.48	9.81	75.64	positive	negative
10	8.74	140	193	2.44	4.38	68.14	positive	negative
11	6.38	147	270	/	5.68	72.07	positive	negative
12	11.11	158	179	/	4.32	71.91	positive	positive
13	13.14	144	71	5.42	5.99	61.18	positive	positive
14	5.38	136	204	2.65	4.63	69.93	positive	negative
15	10.85	83	262	4.34	6.04	60.38	negative	negative
16	5.53	120	150	2.68	4.99	67.83	positive	positive
17	6.59	119	201	4.01	3.21	68.23	positive	positive
18	7.00	123	407	3.25	4.68	76.72	positive	negative
19	7.64	142	332	3.12	4.09	50.00	negative	negative
20	8.91	133	2237	4.2	7.54	66.49	positive	negative
21	6.61	128	270	2.17	4.71	65.38	negative	negative
22	11.15	120	160	4.26	7.97	52.40	positive	negative
23	3.85	109	334	3.91	5.88	55.19	positive	negative
24	14.58	135	214	5.98	9.04	70.72	positive	negative
25	10.71	161	152	4.01	7.17	69.64	positive	negative
26	10.14	158	204	4.52	5.7	93.58	positive	negative

^*^ / = data unavailable; Abbreviations: SARS-CoV-2 = severe acute respiratory syndrome coronavirus type 2

**Table 6:** CSF characteristics of patients with COVID-19 infection only and co-existing other infections.

Variable	COVID-19 infection only (n = 15)	Co-existing other infections (n =8)	*P* value
Elevated CSF pressure, n (%)	5 (33.3)	2 (25.0)	0.679
Elevated leukocyte count, n (%)	2 (13.3)	3 (37.5)	0.181
Elevated total protein, n (%)	7 (46.7)	4 (50.0)	0.879
Positive CSF-IgG, n (%)	3 (20.0)	1 (12.5)	0.651
Blood-CSF barrier dysfunction^a^, n (%)	8 (53.3)	4 (50.0)	0.879

^a^Blood-CSF barrier dysfunction was defined as QAlb > Qlim (Alb) Abbreviations: CSF = cerebrospinal fluid

## Abbreviations

COVID-19: coronavirus disease 2019
CSF: cerebrospinal fluid
IQR: interquatile range
qRT-PCR: quantitative reverse transcriptase polymerase chain reaction
OCB: oligoclonal band
SARS-CoV-2: severe acute respiratory syndrome coronavirus type 2
